# Wastewater Measures of SARS-CoV-2 Accurately Predict Frequency of Symptomatic Infections in the Community

**DOI:** 10.1093/infdis/jiaf242

**Published:** 2025-07-24

**Authors:** Charles R Doss, Mark J Osborn, Stacey Stark, Joshua Rhein, Jacalynn Donkersgoed, Donna Budde, Shannon Champeau, Carolyn Meyer, Mason Hayden, Laura Landini, Difan Ouyang, Lappui Chung, Yi Tang, Sara Vetter, Timothy W Schacker

**Affiliations:** School of Statistics, University of Minnesota, Minneapolis, Minnesota; Department of Pediatrics, University of Minnesota, Minneapolis, Minnesota; U-Spatial, Research Computing, University of Minnesota, Duluth, Minnesota; Department of Medicine, University of Minnesota, Minneapolis, Minnesota; Office of Employee Occupational Health and Safety, Fairview Health Services, Minneapolis, Minnesota; Office of Employee Occupational Health and Safety, Fairview Health Services, Minneapolis, Minnesota; Department of Pediatrics, University of Minnesota, Minneapolis, Minnesota; Department of Pediatrics, University of Minnesota, Minneapolis, Minnesota; Department of Pediatrics, University of Minnesota, Minneapolis, Minnesota; Department of Pediatrics, University of Minnesota, Minneapolis, Minnesota; School of Statistics, University of Minnesota, Minneapolis, Minnesota; School of Statistics, University of Minnesota, Minneapolis, Minnesota; School of Public Administration, University of New Mexico, Albuquerque, New Mexico; Minnesota Department of Public Health, Saint Paul, Minnesota; Department of Medicine, University of Minnesota, Minneapolis, Minnesota

**Keywords:** COVID-19, SARS-CoV-2, wastewater, predictive model, polymerase chain reaction

## Abstract

**Background:**

Widespread immunity through vaccination or natural infection has altered the predictive ability of wastewater for hospitalization and mortality.

**Methods:**

Between January 2022 and August 2024, we conducted a longitudinal observational study that aimed to examine the correlation between symptomatic COVID-19 in health care employees and SARS-CoV-2 wastewater community levels. Wastewater was analyzed by quantitative reverse transcription polymerase chain reaction for detection of SARS-CoV-2. The Fairview Employee Occupational Health office provided deidentified data.

**Results:**

We collected 215 wastewater samples from the Twin Cities Wastewater Treatment Plant over a 32-month interval. Over that period, there were 6879 positive SARS-CoV-2 test results reported to Fairview Employee Health from individuals who lived in the wastewater catchment area. We found that SARS-CoV-2 levels in wastewater accurately predicted the subsequent COVID-19 case count the following week in the community (*P* = .001).

**Conclusions:**

These data demonstrate the utility of SARS-CoV-2 wastewater surveillance as it accurately predicts the frequency of symptomatic infection in the community.

## Teaser

SARS-CoV-2 levels in wastewater accurately predicted symptomatic COVID-19 cases among health care workers approximately a week in advance, highlighting wastewater surveillance as a powerful tool for monitoring community transmission of viral diseases in the contemporary immune landscape.

Measuring wastewater for polio has been used for several decades to monitor for outbreaks [1, 2], and it has subsequently been used as a method for monitoring for antibiotic resistance [3], the presence of illicit drugs [4], or environmental contaminants [5]. Testing wastewater for SARS-CoV-2 emerged early in the COVID-19 pandemic as a public health tool to monitor virus levels circulating in the community, and we and others showed that it successfully provided a 2-week predictor of surges in hospitalizations [6–9].

In the early stage of the COVID-19 pandemic (2020–2022), levels of SARS-CoV-2 in wastewater could be used as a leading indicator for an upcoming surge in hospitalizations [10–12]. However, as the pandemic evolved, this relationship degraded, and in Minnesota virus levels in wastewater now appear to be a lagging indicator of hospitalizations [13]. Fewer people with symptomatic COVID-19 require hospitalization because of preexisting immunity from vaccination or natural infection, although they contribute to virus levels in the wastewater. In addition, most testing is done at home, and results are less often officially reported to public health agencies. As a result, monitoring COVID-19–related illness is more difficult and less precise. Determining epidemiologic trends of virus spread and the burden of disease at the community level has become challenging. Tracking the spread of infection throughout the community as well as the emergence of new variants remains important. As such, increasing public awareness can help health care officials and people at risk for severe COVID-19 take appropriate precautions. In addition, knowledge of an upcoming surge in employee illness could be important in planning for workforce shortages due to illnesses such as COVID-19. This is particularly important in the health care setting, where staffing remains tight, but also in any organization with a large workforce. To that end, we hypothesized that there would be a significant correlation between symptomatic COVID-19 and SARS-CoV-2 levels in wastewater. We partnered with Fairview Health Services, a large health care system in Minnesota. Employees who test positive for COVID-19 are required to show proof of the positive test result to be excused from work and be absent from work until the infection has resolved. We compared the frequency of symptomatic COVID-19 illness in Fairview Health Services employees who live in the sewered area that we sampled with the amount of SARS-CoV-2 detected in wastewater.

## METHODS

### Study Oversight

The University of Minnesota institutional review board reviewed the project and determined that this research study did not meet the criteria for human research (UMN IRB 00023171).

### Employee Health Data

Data on the deidentified test results and the city of residence were provided by the Employee Occupational Health and Safety office at Fairview Health Services. If the city of residence of the person with a positive SARS-CoV-2 test result was within the catchment area of the Twin Cities Wastewater Treatment Plant (TCWWTP) and was not served by another wastewater treatment plant according to the Metropolitan Council Regional Planning board [14], the individual was included in the study. All other individuals were excluded. The raw dataset included 13 909 positive test results, and 6879 of those corresponded to the TCWWTP and are included in the study.

### Laboratory Measures of SARS-CoV-2 in Wastewater

**Figure 1.**
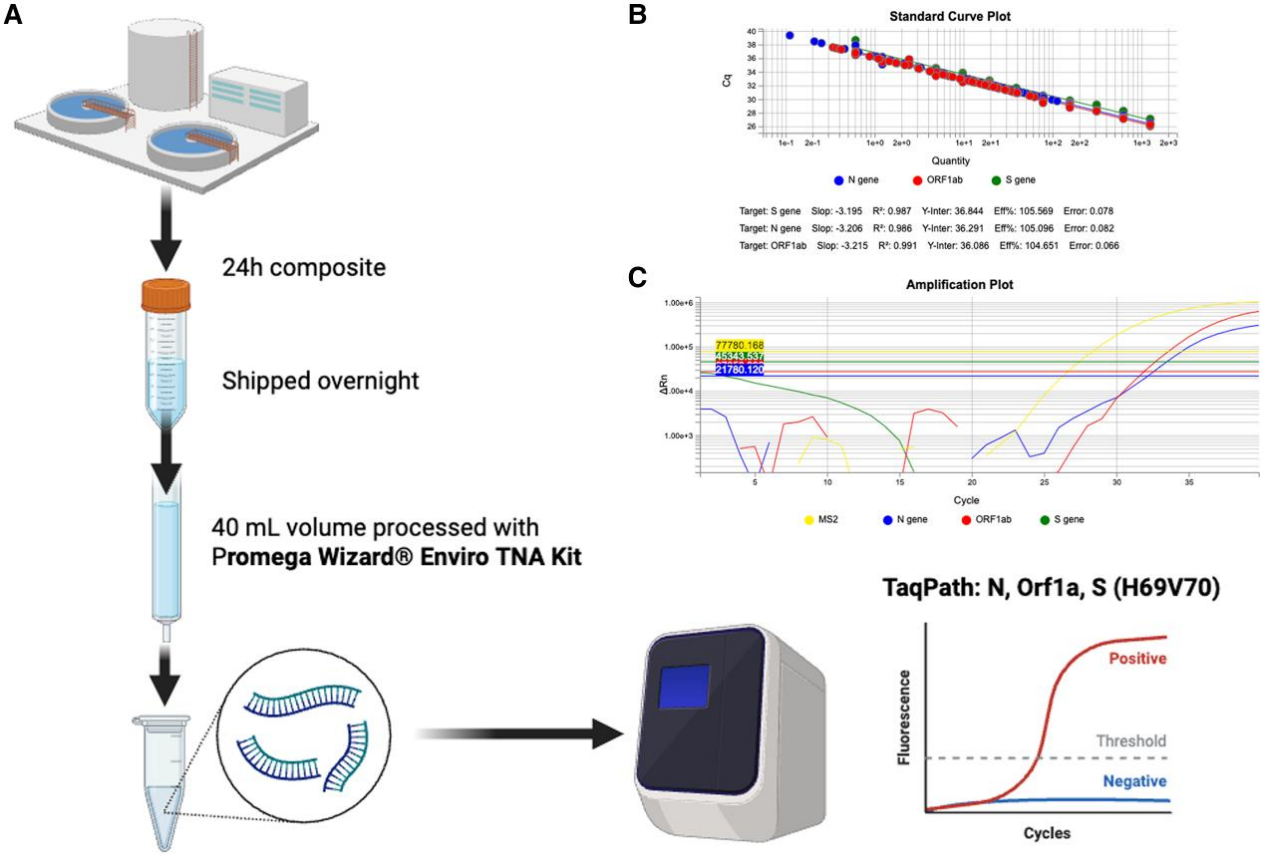
Sample collection and analysis. *A*, Influent was collected and shipped for isolation of total nucleic acids that were analyzed by qRT-PCR. *B*, Representative qRT-PCR standard curve with known quantities of SARS-CoV-2 for copy number assessment. *C*, Amplification plots of a wastewater sample with a spike in control MS2 (yellow), Orf1ab (open reading frame 1ab; red), N (nucleocapsid; blue), and S (spike; green). qRT-PCR, quantitative reverse transcription polymerase chain reaction.

Fifty milliliters of 24-hour composite influent samples were obtained from the TCWWTP and shipped by overnight courier on ice. Upon receipt, 40 mL of the sample was pasteurized at 60 °C for 1 hour and then filtered through a 0.22-µm Sartorius filter station. After filtering, 50 µL of the MS2 Phage Control Standard from the TaqPath COVID-19 Combo Kit (Thermo Fisher) was added, and total nucleic acid was isolated by the Promega Wizard Enviro TNA Extraction Kit. Nucleic acid (5 µL) was used in duplicate for SARS-CoV-2 quantitative reverse transcription polymerase chain reaction (PCR) with the TaqPath COVID-19 Combo Kit (Thermo Fisher) for simultaneous detection of the MS2 phage control and SARS-CoV-2 nucleocapsid (N), open reading frame 1ab (Orf1ab), and spike (S) genes. PCR was performed on the QuantStudio 5 Real-Time PCR instrument (Thermo Fisher) in a 384-well format. Copy number was calculated from standard controls that were employed in serial dilutions from 2500 to 0.5 total copies of nucleic acid. Analysis was performed by Real-Time PCR System Software (Design and Analysis version 2.6.0; Applied Biosystems, Thermo Fisher). Data were reported as copies of SARS-CoV-2 gene target per liter of wastewater influent (Figure 1). The S gene probe binds across amino acids 69 and 70, which are occupied by histidine and valine, respectively. This hypervariable region can be either present, resulting in an S gene signal, or absent, leading to an S gene target failure, depending on the genome sequence of a given variant. A noisy signal on the S gene amplification plot can be seen until approximately cycle 20, which does not generate a bona fide amplification product and correlates to the 69–70del in the S gene, resulting in S gene dropout in the reverse transcription PCR assay.

### Statistical Methods

We used the copy number per liter of the Orf1ab protein gene detected in the sample for our statistical model. Prior work suggests using the Orf1ab protein rather than the N protein [15]. In addition, the daily flow volume of wastewater was provided by the TCWWTP. We multiplied each wastewater RNA concentration measurement by the daily flow volume. Then we aggregated all wastewater measurements in a week (Sunday–Saturday) into a single weekly average measurement of the wastewater SARS-CoV-2 RNA level. If there was a single measurement in a week (14 such weeks), it was used directly as the week's measurement. If there were no measurements in a week (6 such weeks), the measurement was treated as missing in our statistical model.

Prior work on population modeling of outcomes from COVID-19 might be segmented into 4 approaches with some overlap. The most direct and common approach is to focus on linear models (eg, studying the correlation coefficient between wastewater and COVID-19 infections), which are well illustrated in a meta-analysis by Li and colleagues [16]. One main benefit is that such techniques are extremely well understood and also are the easiest to interpret. Other works are based on so-called state-space models. In these, the underlying population infection levels are not assumed to be observed directly; rather, some mathematical mechanisms are specified that govern how population infection levels are related to observable levels (eg, wastewater, infection, hospitalization, death) [17]. These models are more sophisticated than direct linear modeling approaches but thus require more expertise and explanation to understand and interpret, and they can sometimes have unforeseen modes of failure due to their complicated nature. A third approach is to use so-called epidemiologic compartment models (susceptible-infected-recovered [SIR] type); sometimes these are placed within a state-space format [18]. The benefit to these compartment models is the possibility of making medium- or even long-term predictions (rather than just short-term ones), which is not realistically possible without a mathematical model of how infection dynamics progress over time (something that the other 2 approaches do not commonly include). The concern, however, is that unless the conditions of the epidemiologic model are satisfied and do not change over time, the predictions can be poor, and in reality, population conditions are constantly changing so this is often unrealistic. A fourth approach, broadly speaking, is to use flexible machine learning methods for prediction. Yet, these are typically difficult to interpret, and so we do not focus on them here. Chen and colleagues provide a broad survey of the literature that covers correlation analysis, linear (autoregressive moving average [ARMA]) time series models, compartment models, and machine learning methods [19].

In this article, we focus on the first approach, based on direct linear models. As mentioned, they are the simplest to interpret and best understood by the scientific community. Since our goal is to demonstrate the clear value that wastewater has in the current population immunologic landscape and to show that wastewater continues to have a strong direct relationship to infection cases (at least symptomatic ones) that are generally no longer observed for US populations, ease of interpretation is of key importance. We will develop and study the use of state-space models in a separate article.

We implemented 2 models to determine the ability of measures of SARS-CoV-2 in wastewater to predict future clinical cases of COVID-19. Both models used logged case count data as the response variable. In model 1 we included case count and wastewater data from the previous week as covariates, and in model 2 we included only wastewater data as a covariate.

For model 1 we used a time series linear regression model to model the relationship between levels of SARS-CoV-2 in wastewater and frequency of employee absence in the same interval. We fit an autoregressive model with 2 lags (a second-order autoregressive model or AR(2)) to the logged case count series *C_t* and included 2 lags of the logged wastewater series as covariates in a linear regression. That is, letting *C_t* be the log_10_ case count at week *t* and letting *W_t* be the log_10_ wastewater Orf1ab target copies per liter in week *t*, we specified *C_t* = *a*0 + *a*1 C_{*t* – 1} + *a*2 *C*_{*t* – 2} + *b*1 *W*_{*t* – 1} + *b*2 *W*_{*t* – 2} + noise_*t*, where noise_*t* is modeled as independent and identically distributed noise. Our model-fitting method was robust to minor heteroscedasticity caused by the small number of weeks with only 1 measurement instead of 2 [20]. This model was selected by minimizing the bayesian information criterion [20] over ARMA(*p*,*q*) models (*p* ≤ 3, *q* ≤ 3) and the number of lags (0–4) of wastewater to include. Minimizing the corrected Akaike information criterion [20] yields the same model. Fitting of ARMA models was accomplished by the normal maximum likelihood method [20] with the sarima() function in the astsa package (version 2.1) in R programming language (version 4.4.1).

Model 2 is a linear regression model [21] that excludes the auto-lags of *C_t* on itself. That is, we fit *C_t* = *a*_0 + *b*1 *W*_{*t* – 1} + noise_*t* with independent and identically distributed noise_*t* variables. This model had the smallest bayesian information criterion value over all models with 0 to 5 lags of the wastewater covariates. Fitting was accomplished by the lm() function in the R programming language (version 4.4.1).

## RESULTS

**Figure 2.**
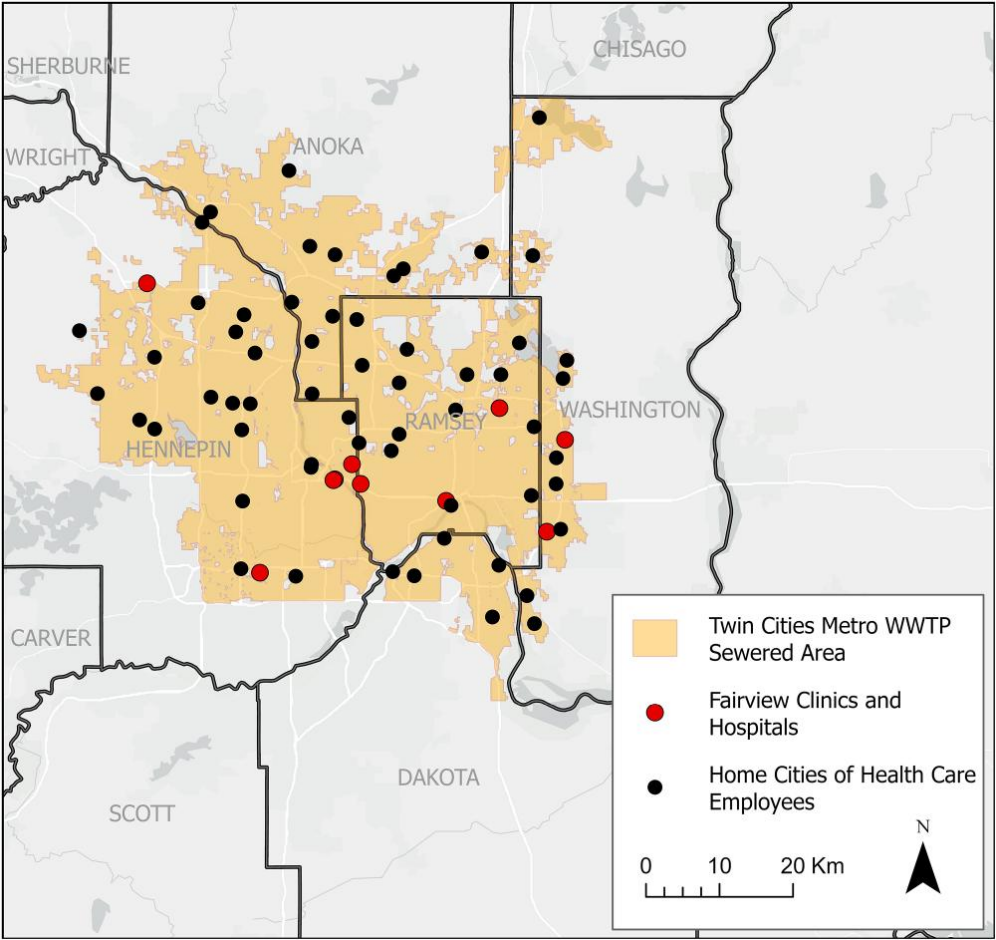
Map of the wastewater collection area (yellow) for the Minneapolis and Saint Paul metropolitan area. Clinical sites are indicated by red dots, and each black dot represents a city where a Fairview Health employee included in the study resided. Abbreviation: WWTP, Wastewater Treatment Plant.

Between January 2022 and August 2024, we collected 215 wastewater samples from the TCWWTP in Saint Paul, Minnesota (Figure 2). This facility collects wastewater from a significant portion of the 7-county metropolitan area and samples approximately 1.85 million people [14]. The overall population of the 7-county metropolitan area is 3.2 million people. Therefore, we sampled approximately 57% of the total people living in the Twin Cities metropolitan area.

M Health Fairview is a large health care system in the state of Minnesota [22] that is headquartered in Minneapolis, and it employs approximately 34 000 individuals. All non–remote work Fairview Health employees must present evidence of a positive COVID-19 test result prior to being excused from work for purposes of home isolation. Acceptable tests included laboratory-based reverse transcription PCR or home-based antigen testing by lateral flow assay. The Fairview Employee Occupational Health and Safety office verified positive test results by photograph or written proof of laboratory testing. A positive result on a given day indicates that an individual has reported a test taken on that day and would be out of work starting on either that day or the next. These data were collated for all of the Fairview Health clinics and hospitals operating within the sewershed district.

**Figure 3.**
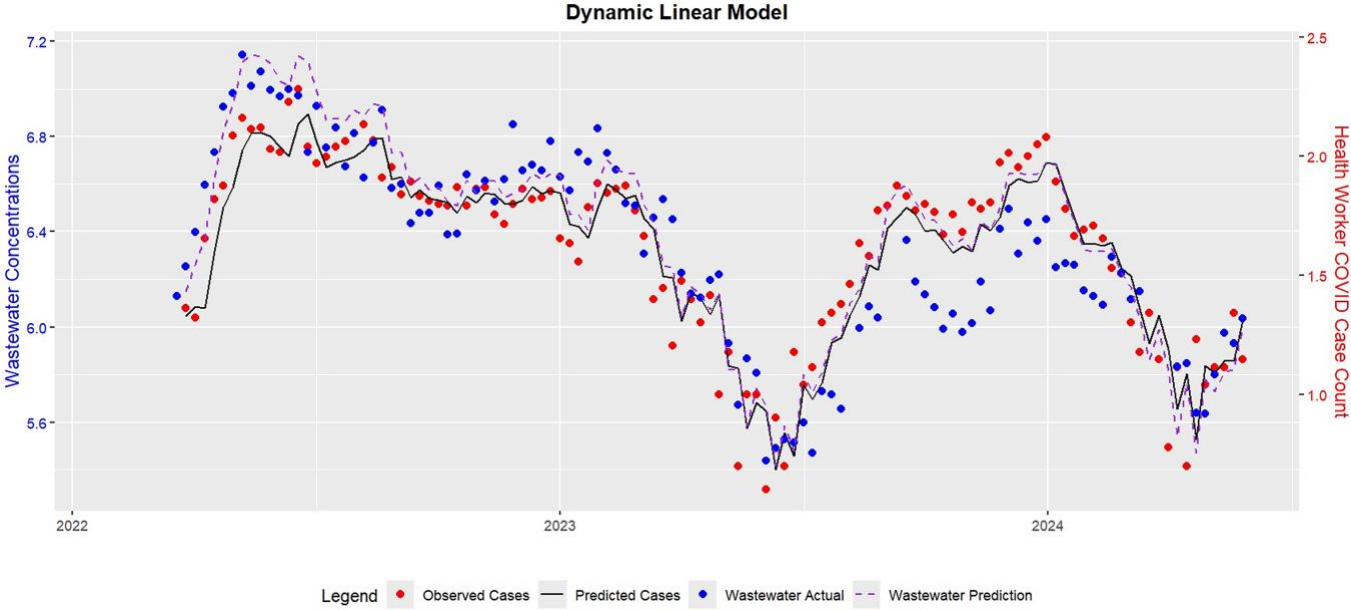
Relationship between employee absence with symptomatic COVID-19 infection and levels of SARS-CoV-2 in wastewater from the same area. The red circles represent the frequency of cases reported in that weekly interval, and the blue circles represent the number of copies of SARS-CoV-2 per liter of wastewater measured in that same interval. The dotted line is the prediction from model 1.

We show in Figure 3 the analysis comparing SARS-CoV-2 levels in wastewater and the frequency of known symptomatic COVID-19 cases from employees who lived in the area served by the TCWWTP. In the 32 months of our study, we observed 3 distinct surges in case counts and correlative levels of virus in wastewater. The first began in January 2022 with a rapid increase in concentrations that did not resolve until the spring of 2023. The second was in July 2023 and the third in June 2024. Each surge was characterized by a rapid increase in case counts and quantity of virus in wastewater.

For model 1, which incorporates the case counts and the measures of virus in wastewater, the fitted coefficients (*P* values) are *a*1 = 0.53 (<.001), *a*2 = 0.37 (<.001), *a*0 = −0.12 (.14), *b*1 = 0.33 (.001), and *b*2 = 0.11 (.28). The fact that *b*1 has a very small *P* value shows that wastewater is statistically significant for predicting positive case counts in the following week. Furthermore, fitting the same model but excluding the wastewater terms yields a 13% increase in the error standard deviation estimate. That is, the wastewater data have information that supplements the information available in past case count data, and the wastewater measurement coefficients are statistically significant predictors for predicting the case counts in the concurrent week.

For model 2, which uses only measures of virus in wastewater as the covariate, the estimated coefficient (*P* value) is 0.71 (<.001), but more important, the coefficient of determination *R*^2^ is 0.62, meaning that 62% of the variation in the case counts can be explained by the wastewater data. *R*^2^ is the square of the Pearson correlation coefficient, and its high value demonstrates the correlation between *W*_{*t* – 1} and *C_t* and that measures of SARS-CoV-2 in wastewater can accurately predict case counts 1 week ahead.

Both models show that wastewater surveillance measurements predict case counts for the following week. Model 1 incorporates all of the data that we have (ie, wastewater and concurrent case counts) to predict future case counts; however, the clinical utility of this approach is limited as concurrent accurate case count data are typically not readily available. Model 2, though, relies only on wastewater measures of virus and still shows very high predictive ability for cases the following week.

## DISCUSSION

Our hypothesis was that measures of SARS-CoV-2 in wastewater would accurately predict the burden of symptomatic COVID-19 in the community. We used employee health data from a large health care system in the Twin Cities metropolitan area, where absence from work due to COVID-19 required proof of a positive test result, to compare with measures of virus in wastewater from the same geographic region. We were able to match individual workers to the city in which they lived, and we used data only from people who lived in the sewershed that we were sampling. These data unequivocally demonstrate that levels of SARS-CoV-2 in wastewater predict the frequency of symptomatic COVID-19 in the community approximately a week in advance of the clinical cases.

We noted that over the 32-month interval that data were collected for this study, there were 3 surges of virus activity (Figure 3), which began with a rapid increase of virus in wastewater and clinical cases during the summer months and persisted throughout the winter, followed by a rapid decrease in the spring. If this pattern continues, it suggests that SARS-CoV-2 may not have the same seasonal patterns as other respiratory viruses, such as influenza or respiratory syncytial virus, which increase during the fall and winter months. It is possible that this reflects the unique transmission dynamics of SARS-CoV-2 over the study period, including the emergence of novel variants and shifts in population immunity and behavior. It is also unusual that the 3 periods of rapid increase observed in this study occurred during the time of the year when most people in Minnesota spend more time outdoors and children are not in school, as we would not expect rapid transmission of a respiratory virus under those circumstances. If the first 2 surges were any indicator, it is likely that cases in the Twin Cities will continue to increase through the fall months and not begin to decline until spring. While our study provides meaningful insight into temporal trends in wastewater viral levels and clinical case counts, our study duration is insufficient to make firm conclusions around seasonality. Ongoing surveillance is essential to evaluate whether wastewater monitoring remains predictive across seasonal contexts.

Importantly, this surveillance technique could be expanded to target infectious diseases such as H5N1 influenza [23] or mpox [24], and application of next-generation sequencing to wastewater could identify unexpected pathogens circulating in the community. Sequencing samples to identify microbes has been done, and informatics pipelines to support these types of analyses are being developed [25].

There are limitations to the interpretation of these data. This study aimed to evaluate the correlation between wastewater SARS-CoV-2 levels and a well-defined, systematically tested population in a single metropolitan area. Population movement can alter viral levels in wastewater, potentially overestimating infection burdens in mobile populations (eg, tourists, commuters) [26]. The Twin Cities' relatively stable population limits this concern, though our findings may not generalize to more mobile areas. Our clinical data are from working-age adults only and are not representative of the general population, including children and older adults, who may experience different infection rates and disease severity. We also do not know the SARS-CoV-2 infection rate in children or older adults, who are generally considered more susceptible to severe COVID-19. Our data come from health care workers, who would be more likely to have multiple exposures in the course of their work, and we acknowledge that this could overestimate transmission rates in the general community. We also were not able to account for people reporting more than 1 positive test result because all of the results were deidentified. Nevertheless, the high degree of correlation and the predictive ability and value of wastewater measures reduce the impact of these limitations.

Collectively, these data demonstrate that wastewater can be an important and useful tool to track the spread of clinical COVID-19 disease through a community, which has important implications for public health. Wastewater surveillance also can provide an important tool for industry and corporations making workforce decisions around flexing capacity during periods showing significant increases in viral load. This is of acute importance in sectors already experiencing shortages, as in health care settings, where wastewater data could inform resource allocation by guiding targeted clinical testing and enabling early implementation of infection prevention strategies. For individuals, surveillance indicators can be useful when making decisions about travel, vaccinations, and social activities in general, though translating wastewater surveillance trends into actionable public health interventions requires further exploration. Future work should focus on integrating wastewater surveillance with other epidemiologic data sources to develop real-time decision-making frameworks that support public health responses to emerging outbreaks.
